# Primo Vascular System: Past, Present, and Future

**DOI:** 10.1155/2013/240168

**Published:** 2013-09-22

**Authors:** Byung-Cheon Lee, Walter J. Akers, Xianghong Jing, M. Isabel Miguel Perez, Yeonhee Ryu

**Affiliations:** ^1^Ki Primo Research Laboratory, Division of Electrical Engineering, KAIST Institute for Information Technology Convergence, Korea Advanced Institute of Science and Technology (KAIST), Daejeon 305-701, Republic of Korea; ^2^Department of Radiology, Washington University School of Medicine, St. Louis, MO 63110, USA; ^3^Institute of Acupuncture and Moxibustion, China Academy of Chinese Medical Sciences, Beijing 100700, China; ^4^Unit of Human Anatomy and Embryology, University of Barcelona, Casanova, 143 08036 Barcelona, Spain; ^5^Acupuncture, Moxibustion & Meridian Research Group, Medical Research Division, Korea Institute of Oriental Medicine, Daejeon 305-811, Republic of Korea

What is Primo Vascular System (PVS)? Let us take a journey through oriental medicine in a time machine. Over the past 2000 years, acupuncture and moxibustion in Chinese medicine have been developed based on the concept of the meridian system; however, the anatomical reality of the meridian system has been controversial in various aspects. Even today, the meridian system is still being investigated with well-known anatomical structures. Among them, connective tissues called the fascia system are representative ones for which the putative function of the meridian system has been established and is understood [1].

A fundamental insight into the acupuncture meridian system and its novel anatomical structures was conceived by Kim in the 1960s [2]. According to his idea, the meridian system has the role of circulating DNA microparticles, named “Signals,” with several hormones independently from the cardiovascular and the lymph systems. In the 1970s, Fujiwara tried to duplicate and verify Kim's findings; however, his works have also been neglected [3]. Since 2002, Soh's group at Seoul National University, Republic of Korea, has tried to verify the findings of Bonghan Kim's work, and they found much evidence suggesting that Bonghan Kim's ideas on the acupuncture meridian system are reasonable [4]. 

At present, what do we study about the PVS? to answer this question and for insight into current works on this novel system, we have a new special issue, Primo Vascular System, in which we have published several research papers and a few review articles. The research papers can be classified as those directly related to the function of PVS and those focusing on the discovery of this new PVS. Representative articles in the former category are the C. H. Leem group's stem cell work *“Expression of stem cell markers in primo vessel of rat,”* and K.-S. Soh group's endothelial cell work *“Discovery of endothelium and mesenchymal properties of primo vessels in the mesentery.”* In the latter category are the works of Y. H. Ryu laboratory *“Primo vascular system accompanying a blood vessel from tumor tissue and a method to distinguish it from the blood or the lymph system”* S. Z. Yoon's medical team *“Composition of the extracellular matrix of lymphatic novel threadlike structures: is it keratin?”* and B.-C. Lee's laboratory *“Evidence for the primo vascular system above the epicardia of rat hearts.”* Also, a Chinese team led by X. Jing has published a very meaningful idea that the PVS could represent artifacts from pathological conditions *“Preliminary research of relationship between acute peritonitis and celiac primo vessels,”* but another Chinese team led by W.-B. Zhang has suggested via heparin treatment that the PVS might have real anatomical structures *“Study on the formation of novel threadlike structure through intravenous injection of heparin in rats and refined observation in minipigs.”* Based on these data, admittedly, at present, for the PVS to be established absolutely, an international exchange is needed.

On the other hand among controversial research articles, we published a few review papers in which we should pay more attention to two teams, K.-S. Soh's and B. Zhu's (*“50 years of Bong-Han theory and 10 years of primo vascular system”* and *“Historical review about research on “Bonghan system” in China”*). Through their review articles, even beginner in PVS could figure out the overall feature of PVS. Especially for the research history of PVS we recommend a review article by Kim [5] in related journal to ECAM, Journal of Acupuncture and Meridian Studies. We also suggest researchers to pay some attention to two review articles: one is J. Jeon's approach on the relationship between the history of acupuncture meridian system and PVS *“The meanings and prospects of primo vascular system from the viewpoint of historical context.”* The other is novel insight into the function of PVS as DNA particles circulation system suggested by S. Z. Yoon *“Toward a theory of the primo vascular system: a hypothetical circulatory system at the subcellular level.”*

In the future, what will we study about the PVS? Based on our long research careers, we editors suggest the following areas of study to establish an international unified consensus for the novel system that is called the Primo Vascular System:

1. the establishment of the Primo Vascular System in terms of a novel circulation system,
2. the concept of “Sanal” and the relationship between stem cells and Sanals,
3. the involvement of Sanals in cancer metastasis,
4. The potential of the PVS in the brain for diagnosing and treating degenerative brain diseases.

Now let us think of “time” by leaving the time machine. Time really flows in only one direction, toward the future! Thus, our research minds should be directed beyond the past and the present and toward the future. With free, dedicated efforts toward human-oriented holistic medicine, we should be able to build a real evidence-based alternative medicine. Given the present circumstances, a new circulation concept, the Primo Vascular System, is waiting for us to establish fully its potential for benefitting all mankind in ways not previously known.

*Byung-Cheon Lee* *Walter J. Akers* *Xianghong Jing* *M. Isabel Miguel Perez* *Yeonhee Ryu*
